# POSTER ABSTRACTS *from* Third Annual Public Meeting: Mobilizing Computable Biomedical Knowledge (MCBK 2020)

**DOI:** 10.1002/lrh2.10256

**Published:** 2020-12-20

## Technical Posters

## An automated process to catalog and track COVID‐19 public health guidance

### Peter Taber^1^, Elisa Rocha^2^, Guilherme Del Fiol^3^, Catherine J. Staes^4^, Adria Lam^5^, Saifon Phengphoo^4^, Saverio M. Maviglia^6^, Roberto A. Rocha^6^

#### ^1^ VA Salt Lake City Health Care System, Salt Lake City, Utah; ^2^University of Massachusetts Medical School, Worcester, Massachusetts; ^3^Department of Biomedical Informatics, University of Utah, Salt Lake City, Utah; ^4^College of Nursing University of Utah, Salt Lake City, Utah; ^5^Albany Medical College, Albany, New York; ^6^Brigham and Women's Hospital, Harvard Medical School, Boston, Massachusetts

Numerous and constantly changing online information sources provide public health (PH) guidance about COVID‐19. Systematically inventorying online guidance is critical to understand what information is available, from where, and how it changes over time. We implemented an automated process to identify online COVID‐19 guidance resources (URLs). The structures of websites of state PH agencies for Florida (FL), Illinois (IL), Massachusetts (MA) and Utah (UT) were discovered using a commercial Web crawler. URLs identified via Web crawler were processed by scripts in Apple Automator to extract links to CDC resources from webpages and static documents. Inventoried URLs were screened for relevance. The automated process produced an inventory of guidance resources owned by a state PH authority; and an inventory of external guidance resources referenced by the state PH authority. Number of state‐maintained COVID‐19‐related URLs was 54 for FL; 216 for IL; 581 for MA; and 229 for UT. Of total URLs, guidance documents constituted 57% for FL; 51% for IL; 55% for MA; and 32% for UT. A random sample of guidance documents (n = 234) was drawn to support the development of a preliminary taxonomy of COVID‐19 PH guidance.

## Bridging the gap between patient data and clinical situations and knowledge through a searchable clinical concept glossary

### Jane Shellum^1^, Marc L. Sainvil^1^, Adam Bartscher^1^, Sottara Davide^1^

#### ^1^Mayo Clinic, Rochester, Minnesota

This poster presentation is particularly relevant to knowledge management professionals. The Clinical Concept Glossary (CCG) exposes the (i) semantic and (ii) operational definitions of resolvable clinical concepts, in the form of “Glossary Entries”. An operational definition is a computable piece of knowledge that is either implemented in a Computable Biomedical Knowledge (CBK) artifact, or is deployed as a service for which a computable API specification is published. The CCG itself consists in a knowledge base of semantically annotated computable metadata records, an API for programmatic consumption, and an end‐user application that facilitates search, consultation and retrieval. The Clinical Concept Glossary allows to centralize the (computational) methods that can be leverage to answer clinically relevant questions using patient data. As different teams across various projects aim to solve similar questions, there is a significant risk of misusing developers and clinician time, duplicative efforts, inconsistent definitions, and sharable definitions. The CCG presents an opportunity to increase the value of Mayo Clinic's investment in health information technology by improving accuracy and consistency of clinical applications, analytics, and research cohorts while reducing the time and expense of application development.

## CBK of physiologic time‐series data—The Sickbay platform

### Bikram Day^1^, Raajen Patel^1^

#### ^1^Medical Informatics Corporation, Houston, Texas

We present Sickbay, an FDA‐cleared software platform for remote patient monitoring and alarm distribution. It passively records time‐synchronized waveforms, vitals, alarms, and settings from bedside medical devices along with labs, medications and observations. It then stores that high‐fidelity data at native resolution, in an open, computable format without constraints on duration. To utilize this data fully, researchers can use included APIs and an SDK to develop and deploy CBK at scale, enabling a rapid virtuous cycle of research insight, validation, and clinical practice.

## CDS connect—A platform for sharing and authoring CDS artifacts

### Lacy Fabian^1^, Chris Moesel^1^, Shafa Al‐Showk ^2^, Roland Gamache^2^, Edwin A. Lomotan^2^

#### ^1^The MITRE Corporation, McLean, VA; ^2^Agency for Healthcare Research and Quality, Rockville, MD

CDS Connect is an open‐source platform for sharing and authoring clinical decision support (CDS) artifacts across myriad health domains that is part of the Agency for Healthcare Research and Quality's CDS initiative. CDS Connect assists clinicians and provider organizations, health information technology vendors and federal health research organizations in translating evidence‐based knowledge into implementable clinical tools. The components of CDS Connect include a repository of CDS artifacts, an authoring tool for creation of CDS, and prototype development tools for testing and integrating CDS into health systems. CDS Connect's platform is designed to encourage members of the CDS community to contribute their CDS artifacts to the repository thereby promoting the translation of evidence‐based research into clinical practice. Individuals who would like to learn more about contributing or other ways to engage can visit https://cds.ahrq.gov/cdsconnect.

## Executable search notebooks on the PATTIE platform

### Michael Segundo Ortiz^1^, Javed Mostafa^2^, Elliot Hauser^3^

#### ^1^Carolina Health Informatics Program, University of North Carolina at Chapel Hill, Chapel Hill, North Carolina; ^2^Carolina Health Informatics Program (CHIP), School of Information and Library Science, Biomedical Research Imaging Center Director, University of North Carolina at Chapel Hill, Chapel Hill, North Carolina; ^3^School of Information, University of Texas at Austin, Austin, Texas

This technical poster demonstration is particularly relevant to voice‐enabled biomedical information retrieval from a prototype system called Publication Access Through Tiered Interaction & Exploration (PATTIE).^1^ The current system version proposed for MCBK 2020 seeks to demonstrate a completely novel paradigm for information retrieval ‐ search notebooks. By interacting with one's voice through a minimal dictionary language (ie, few commands), PATTIE records and organizes a transcript of the search strategy into strata such as voice commands, intentions, thoughts, and citations. The strata are serialized in object notation format which allows for computationally driven models and extensibility that is limited only by ideation. Currently, we are developing the concept of an executable search notebook that can be shared among project teams during their initial information‐seeking phases within the biomedical literature. We believe this offers benefit, particularly to principal investigators, who often supervise numerous and concurrent projects and would prefer to have a central hub where student and collaborator strategies during the early phases of a research project can be computationally analyzed.

^1^ https://pattie.unc.edu/ .

## Generalizable metadata management for reproducible translational research

### Ramkiran Gouripeddi^1,2,3^, Peter Mo^2^, Richard Bradshaw Mo^2^, Randy Madsen^2^, Le‐Thuy Tran^2^, Ryan Butcher^2^, Mollie Cummins^1,2,3,4^, Katherine Sward^1,2,3,4^, Julio Facelli^1,2,3^

#### ^1^Department of Biomedical Informatics, University of Utah, Salt Lake City, Utah; ^2^Center for Clinical and Translational Science, University of Utah, Salt Lake City, Utah; ^3^Center of Excellence for Exposure Health Informatics, University of Utah, Salt Lake City, Utah; ^4^College of Nursing, University of Utah, Salt Lake City, Utah

Sharing and reuse of biomedical data is critical to enhance research reproducibility and increase efficiency in translational biomedical sciences.^1‐3^ This requires biomedical data and processes to be findable, accessible, interoperable and reusable according to the FAIR guiding principles.^4^ The capture of sufficient metadata is a key requirement for successful data harmonization and integration, knowledge presentation and research process management.

In this presentation we describe a generalizable approach to managing metadata for diverse informatics applications in the translational research spectrum.^5^ OpenFurther's metadata repository^6,7^ (MDR) is an Object Modeling Group specification conformant, FAIR‐compliant standard‐based repository of artifacts and knowledge about things. It stores metadata artifacts and relationships of data and modular components subscribed by OF. These artifacts include, but are not limited to: (1) Logical models, local models, model mappings, (2) Administrative information, (3) Descriptive information, and (4) Translation Programs. These are organized as “Assets” in a custom‐built highly generic and abstracted entity relationship model. Assets may have properties and associations to other assets. Stored metadata is shared in various structured and non‐proprietary formats using translation programs and made available for consumption by different software services.

Considering the data and process complexity within translation research, we conceptually divided metadata management into three categories:

- Data Metadata: Describes the data output resulting from an observation or measurement. This could include sensor measurements, output of computational models, clinical observations, genomic sequence annotations, socio‐behavioral data, and participant report data among others.
- Process Metadata: Describes research or data processes within informatics infrastructure. These include among others sequences of steps followed in different computational models in order to generate outputs, and data transformation and integration workflows to harmonize source data as events or into analytical models. An example of a research process is sensor deployment.
- Knowledge Resources: Metadata that describes the source or instrument used to collect, measure or derive data. These could include sensor devices, electronic medical records or study specific data collection instruments.

We have developed and evaluated the MDR in different technologies including relational,^6^ graph and document stores^8,9^ of different use cases including data federation,^6^ integration,^10^ data quality assessment,^11^ knowledge presentation,^12^ and sensor‐based exposomic research.^8,9^ Future directions include developing methods automate metadata discovery process,^13,14^ consume and store such metadata into the MDR,^15^ maintenance of metadata provenance and trajectory using approaches like blockchain, and data and process orchestration in ultra large scale systems.

This poster presentation is particularly relevant to informaticians developing methods and tools for translational research.

REFERENCES

1. Federer LM, Lu Y‐L, Joubert DJ, Welsh J, Brandys B. Biomedical Data Sharing and Reuse: Attitudes and Practices of Clinical and Scientific Research Staff. PLOS ONE. 2015 Jun 24;10(6):e0129506.

2. Gøtzsche PC. Why we need easy access to all data from all clinical trials and how to accomplish it. Trials. 2011 Nov 23;12(1):249.

3. Ross JS, Lehman R, Gross CP. The Importance of Clinical Trial Data Sharing: Toward More Open Science. Circ Cardiovasc Qual Outcomes. 2012 Mar 1;5(2):238‐40.

4. Wilkinson MD, Dumontier M, Aalbersberg IjJ, Appleton G, Axton M, Baak A, et al. The FAIR Guiding Principles for scientific data management and stewardship. Sci Data. 2016 Mar 15;3:160018.

5. Gouripeddi R, Schultz ND, Bradshaw RL, Mo P, Madsen R, Warner PB, et al. FURTHeR: An Infrastructure for Clinical, Translational and Comparative Effectiveness Research. In: American Medical Informatics Association 2013 Annual Symposium. Washington, D.C.; 2013.

6. Bradshaw RL, Matney S, Livne OE, Bray BE, Mitchell JA, Narus SP. Architecture of a Federated Query Engine for Heterogeneous Resources. AMIA Annu Symp Proc. 2009;2009:70‐4.

7. Mo P, Schultz ND, Bradshaw RL, Butcher R, Gouripeddi R, Warner PB, et al. Real‐time Federated Data Translations using Metadata‐driven XQuery. In: AMIA 2014 Summit on Clinical Research Informatics. San Francisco; 2014.

8. Gouripeddi R, Tran L‐T, Madsen R, Gangadhar T, Mo P, Burnett N, et al. An Architecture for Metadata‐driven Integration of Heterogeneous Sensor and Health Data for Translational Exposomic Research. In: 2019 IEEE EMBS International Conference on Biomedical & Health Informatics (BHI) (IEEE BHI 2019). Chicago, USA; 2019.

9. Sward K, Patwari N, Gouripeddi R, Facelli J. An Infrastructure for Generating Exposomes: Initial Lessons from the Utah PRISMS Platform. In: 2017 International Society of Exposure Science Annual Meeting, Research. Research Triangle Park, NC, USA.; 2017.

10. Gouripeddi R, Warner PB, Mo P, Levin JE, Srivastava R, Shah SS, et al. Federating Clinical Data from Six Pediatric Hospitals: Process and Initial Results for Microbiology from the PHIS+ Consortium. In: AMIA Annual Symposium Proceedings. 2012. p. 281. Available from: http://www.ncbi.nlm.nih.gov/pmc/articles/PMC3540481/.

11. Rajan NS, Gouripeddi R, Facelli JC. A Service Oriented Framework to Assess the Quality of Electronic Health Data for Clinical Research. In: 2013 IEEE International Conference on Healthcare Informatics. 2013. p. 482‐482.

12. Bradshaw RL, Staes C, Del Fiol G, Schultz ND, Narus S, Mitchell J. Going FURTHeR with Metadata Repository. In: Fall 2012 Annual Symposium. Chicago, Illinois; 2012.

13. Wen J, Gouripeddi R, Facelli JC. Metadata Discovery of Heterogeneous Biomedical Datasets Using Token‐Based Features. In: IT Convergence and Security 2017 [Internet]. Springer, Singapore; 2017 [cited 2017 Sep 6]. p. 60‐7. (Lecture Notes in Electrical Engineering). Available from: https://link.springer.com/chapter/10.1007/978-981-10-6451-7_8.

14. Bradshaw RL, Gouripeddi R, Facelli JC. Concept Bag: A New Method for Computing Concept Similarity in Biomedical Data. In: Rojas I, Valenzuela O, Rojas F, Ortuño F, editors. Bioinformatics and Biomedical Engineering. Springer International Publishing; 2019. p. 15‐23. (Lecture Notes in Computer Science).

15. Gouripeddi R, Mo P, Madsen R, Warner P, Butcher R, Wen J, et al. A Framework for Metadata Management and Automated Discovery for Heterogeneous Data Integration. In: 2016 BD2K All Hands Meeting. Bethesda, MD; November 29‐30. Available from: https://zenodo.org/record/167885.

## Linking COVID‐19 guidance for healthcare providers to Ely et al's (2000) taxonomy of generic clinical questions

### Saifon Phengphoo^1^, Peter Taber^2^, Elisa Rocha^3^, Adria Lam^4^, Guilherme Del Fiol^5^, Saverio M. Maviglia^6^, Roberto A. Rocha^6^, Catherine J. Staes^1^

#### ^1^College of Nursing, University of Utah, Salt Lake City, Utah; ^2^ VA Salt Lake City Health Care System, Salt Lake City, Utah; ^3^University of Massachusetts Medical School, Worcester, Massachusetts; ^4^Albany Medical College, Albany, New York; ^5^Department of Biomedical Informatics, University of Utah, Salt Lake City, Utah; ^6^Brigham and Women's Hospital, Harvard Medical School, Semedy, Inc., Boston, Massachusetts

Ely et al (2000) provide a rigorously constructed taxonomy of generic treatment, diagnosis, and other clinical questions likely asked by providers. This taxonomy is useful for organizing clinical content to index for automated retrieval. As a step toward indexing COVID‐19 guidance for healthcare providers, we generated a typology of diagnosis and treatment‐related guidance via inductive qualitative coding of documents retrieved from the CDC, Utah Department of Health and the Massachusetts Department of Public Health. Inductively generated codes were then linked to existing categories in Ely et al's taxonomy. The limited treatment guidance available could be mapped directly to existing taxonomy categories. While some COVID‐19 testing guidance could be mapped directly to the taxonomy, we identified additional content relevant to clinicians being *pushed* by public health authorities that is not yet represented in the taxonomy. Guidance related to test specimen handling and the resumption of elective procedures are two examples of pandemic‐specific clinical guidance absent from the taxonomy. Filling these gaps may help public health improve guidance authoring and developers improve information retrieval tools to better address information needs in the context of a pandemic. *This poster presentation is particularly relevant to researchers interested in knowledge development, indexing and retrieval*.

## Making medication indications computable

### Stuart J. Nelson^1^, Mark S. Tuttle^2^

#### ^1^George Washington University, Washington, District of Columbia; ^2^Apelon, Hartford, Connecticut

This poster is particularly relevant to persons creating interoperable healthcare knowledge artifacts, to those with particular interest in medication information, and those who are investigating organizing contextual healthcare information. Starting with a random sample of 1177 unique medication labels, after exploring the content and several beginning attempts, we represented the sample in a computability‐enabling model. Separating the label into condition, use, and context supports the creation of a disjunct‐of‐conjuncts label model. With this formal model, we could represent the diversity of expressions in the labels, while still hoping to achieve a useful level of consistency. After eliminating 61 labels as those of FDA approved drugs that were not used in therapy or prevention, we were able to formalize 1005 of the labels into 1683 distinct indications for use. The 11 labels not fully formalized, such as those for prednisone and cyclophosphamide, described medications with numerous distinct indications. Only 516 of the products could be represented simply, as just drug and condition without any contextual information. The remainder was more complex. Remaining challenges include representing the extracted information in the standard vocabularies used in EHRs, dealing with mismatches of abstractions, and developing a workflow management system enabling collaborative editing.

## Public health guidance for the COVID‐19 pandemic: A preliminary qualitative analysis

### Saifon Phengphoo^1^, Peter Taber^2^, Elisa Rocha^3^, Adria Lam^4^, Guilherme Del Fiol^5^, Saverio M. Maviglia^6^, Roberto A. Rocha^6^, Catherine J. Staes^1^

#### ^1^College of Nursing, University of Utah, Salt Lake City, Utah; ^2^ VA Salt Lake City Health Care System, Salt Lake City, Utah; ^3^University of Massachusetts Medical School, Worcester, Massachusetts; ^4^Albany Medical College, Albany, New York; ^5^Department of Biomedical Informatics, University of Utah, Salt Lake City, Utah; ^6^Brigham and Women's Hospital, Harvard Medical School, Semedy, Inc., Boston, Massachusetts

Public health guidance during a pandemic targets a wide range of actors and evolves quickly, making it difficult for stakeholders to locate relevant resources in a timely manner. This study aims to test a novel sampling methodology through development of a comprehensive taxonomy for indexing and classifying COVID‐19‐related public health guidance. Guidance resources were sampled from Florida, Illinois, Massachusetts, and Utah state agencies to provide coverage across a broad geographic range and status of COVID‐19 infections. Taxonomy concepts were generated through iterative coding cycles evaluating guidance resources, with independent review by four coders. A consensus process was used to refine code descriptors, provide detailed definitions, and extract illustrative quotations. The resulting taxonomy aims to provide a comprehensive classification according to target stakeholders, settings, topics, and geographical locations, enabling the indexing and retrieval of online public health guidance for COVID‐19. Preliminary results revealed 25 healthcare and non‐healthcare settings and 14 stakeholder roles. Major topic domains include billing, care coordination, disposition and treatment, presentation and diagnosis, reopening process, resource management, and risk management. Ongoing work involves more extensive sampling of healthcare and non‐healthcare related guidance.

## The OpenClinical knowledge model as a template for rapid development of executable clinical guidelines: case study with COVID‐19

### John Fox^1^, Omar Khan^2^, Krishnarajah Nirantharakumar^3^

#### ^1^OpenClinical CIC, London, UK; ^2^University of Warwick, Coventry, UK; ^3^University of Birmingham, Birmingham, UK

Introduction: We report a pathfinder study of AI/knowledge engineering methods to rapidly formalise COVID‐19 guidelines into an executable model of decision making and care pathways. The knowledge source for the study was material published by BMJ Best Practice in March 2020.

Methods: The PROforma guideline modelling language and OpenClinical.net authoring and publishing platform were used to create a data model for care of COVID‐19 patients together with executable models of rules, decisions and plans that interpret patient data and give personalised care advice.

Results: PROforma and OpenClinical.net proved to be an effective combination for rapidly creating the COVID‐19 model; the Pathfinder 1 demonstrator is available for assessment at https://www.openclinical.net/index.php?id=746.

Conclusions: This is believed to be the first use of AI/knowledge engineering methods for disseminating best‐practice in COVID‐19 care. It demonstrates a novel and promising approach to the rapid translation of clinical guidelines into point of care services, and a foundation for rapid learning systems in many areas of healthcare.

## Use of OHDSI cohort definition tool to pilot clinical decision support logic

### Vojtech Huser^1^, Craig Mayer^1^, Nick Williams^1^

#### ^1^Lister Hill National Center for Biomedical Communications, National Library of Medicine, NIH, Bethesda, Maryland

Rule‐based reminders generated by Clinical Decision Support (CDS) modules identify a population of patients that receive a reminder. Such CDS trigger logic is identical or highly related to the concept of executable phenotype definition and further to a set of cohort definition criteria. For the clinical domain of HIV, we describe a project that authors a set of CDS‐focused cohort definitions using Observational Health Data Sciences and Informatics (OHDSI) Atlas tool and tests them on an EHR dataset (from Great Plains Collaborative). The following cohorts were defined (using 12 month span): (1) HIV+ patient with no record of outpatient visit; (2) HIV+ patient with no medication history of anti‐retroviral therapy (ART). We also designed additional cohorts that complement the CDS cohorts: (1) Denominator cohorts assess the overall size of the HIV+ population and (2) compliant cohorts analyze the opposite point of view. The CDS‐themed cohorts are executed over multiple intervals over time. Repeated presence in CDS cohorts is re‐evaluated over a longer 5 year interval (meta CDS cohorts). We found several strengths and weaknesses of the Atlas tool when used for the CDS purpose. This poster presentation is particularly relevant to CDS researchers and developers.

## Using HL7 FHIR and the OMG's business process modeling standards to diagnose and treat pulmonary embolus

### Peter Haug^1,2^, Stanley Huff^1,2^, Joseph Bledsoe^1,2^

#### ^1^Intermountain Healthcare, Salt Lake City, Utah; ^2^University of Utah, Salt Lake City, Utah

We have developed an electronic protocol for diagnosing, risk stratifying, and treating pulmonary embolus. This application illustrates the use of standards‐based tools to implement interoperable software that can support the delivery of complex clinical protocols. It combines the capabilities of standards from the Object Management Group (OMG) and Health Level 7 (HL7). The application was initially developed between 2013 and 2014. At that time, we piloted a working system based on the OMG standard, Business Process Model and Notation, version 2.0 (BPMN 2.0) in four emergency departments in Salt Lake City, Utah. Since then, we have developed a version of the application that implements not only this OMG standard but also uses HL7's Fast Healthcare Interoperability Resources (FHIR) standards for accessing and storing data in a standards‐compliant development sandbox (Logica's simulated EHR). The application has recently been extended to incorporate logic for risk stratification, disposition, and treatment. The user interface is implemented using the Substitutable Medical Applications, Reusable Technologies (SMART) standard for exposing external applications within existing EHR user environments. This poster presentation is particularly relevant to participants who wish to understand the use of standards for interoperability (FHIR and SMART) and knowledge portability (BPMN) to fashion complex clinical applications.

## PROJECT POSTERS

## Computable knowledge base of experimental treatment intervention by disease over time inferred from Clinicaltrials.gov

### Craig Mayer^1^, Nick Williams^1^, Vojtech Huser^1^

#### ^1^Lister Hill National Center for Biomedical Communications, National Library of Medicine, NIH, Bethesda, Maryland

Introduction: Interventional clinical trials registered at ClinicalTrials.gov (CTG) record what interventions are being studied for various conditions. We created a computable biomedical knowledge artifact (regCTG‐disease‐snapshot) that analyzes interventions for diseases over time.

Methods: Originally developed for tracking Covid‐19 trials, our goal is to generalize our analysis of trial interventions to any disease. We identified 4007 MeSH terms and executed the script on each. We implemented a denormalization method to standardize free text interventions (found in CTG) and assigned intervention significance based on counts of trials using given interventions per multiple time units. Due to connections to regulatory approval, we compared results for phase 3 and 4 trials that ended.

Results: Including condition‐intervention combinations with at least two studies, we found 1182 MeSH terms with 13 267 condition‐intervention combinations for Phase 3 compared to 806 conditions with 5965 combinations for Phase 4. We also found that 74.8% of condition‐intervention combinations had all studies reach completed status for Phase 3 compared to 72.1% for Phase 4. The project repository at https://github.com/lhncbc/r-snippets-bmi/tree/master/D-SHOT provides a spreadsheet with all disease‐intervention data and counts in decade increments.

Conclusion: We developed a knowledge base of interventions from trials allowing for monitoring trends of different interventions for given conditions.

## Computing over ophthalmology clinical text from electronic health records: Case study in identifying candidates for low vision rehabilitation using neural word embeddings

### Benjamin Tseng^1^, Sophia Y. Wang^2^

#### ^1^Byers Eye Institute, Stanford University, Palo Alto, California; ^2^Department of Biomedical Data Science, Byers Eye Institute, Stanford University, Palo Alto, California

Background: Low vision rehabilitation improves quality‐of‐life for visually impaired patients, but referral rates fall short of national guidelines. Identifying rehabilitation candidates from electronic health records (EHR) could increase referrals, but most relevant clinical information is in free‐text notes. Neural word embeddings (WEs) enable computing over free text, but general purpose WEs are likely unsuitable for ophthalmology‐specific language. We develop and validate ophthalmology‐specific WEs for identifying vision rehabilitation candidates from the EHR.

Methods: We trained novel ophthalmology‐specific WEs using 138 412 PubMed ophthalmology abstracts and 89 282 ophthalmology EHR notes. PubMed and EHR WEs were compared to general GloVe WEs on two validation tasks: 1) a novel ophthalmology‐domain‐specific analogy test; 2) predicting irreversible vision loss in 5612 low vision patients.

Results: On analogy testing, PubMed WEs scored 95.0% accuracy, outperforming EHR (86.0%) and GloVe (91.0%). On predicting low vision prognosis, PubMed and EHR WEs resulted in similar AUROC (0.830; 0.826), outperforming GloVe (0.778).

Conclusion: We present and validate novel ophthalmology‐specific word embeddings that enable computing over ophthalmology free‐text notes in the EHR. This poster presentation is particularly relevant to those interested in parsing EHR free‐text using biomedical domain‐specific word embeddings to develop predictive models.

## Creating a FHIR resource for communicating statistical models

### Harold P. Lehmann^1^, Abdulrahman M. Alsheikh^1,2^

#### ^1^Division of Health Sciences Informatics, Johns Hopkins University School of Medicine, Baltimore, Maryland; ^2^Department of Public Health, Imam Muhammad Ibn Saud Islamic University, Riyadh, Saudi Arabia

This poster presentation is particularly relevant to building a FHIR resource to enable the communication of statistical models. As part of clinical research, having an on‐demand, seamless data information exchange becomes an urgent concern with the enormous amount of up‐to‐date results that emphasize the need for creating standards to facilitate the communication of statistical models. With the current data standards infrastructure, there is a huge potential for developing a resource for statistical models that allows open access, a better understanding, reproducibility of research results, manipulation and alteration for the resource algorithms.

## Developing new event‐driven CBK network for global real‐time clinical collaborations

### Anjun Chen^1^, Bairong Shen^1^, Rui Zhang^2^, Fei Teng^3^

#### ^1^Institute for Systems Genetics, West China Hospital, Sichuan University, Chengdu, China; ^2^Information Center, West China Hospital, Sichuan University, Chengdu, China; ^3^School of Information Science and Technology, Southwest Jiaotong University, Chengdu, China

This poster presentation is particularly relevant to rapid development of real‐time clinical collaborations across multiple hospitals and/or countries thorough sharing computable biomedical knowledge (CBK) within research network. Rapid dissemination of actionable knowledge is one of the main goals of the learning health system vision. But this goal is severely hindered by the limited international interoperability of clinical knowledge and the lack of ease‐of‐use digital network for global researchers to share new clinical knowledge in real time. To overcome these challenges, this project is developing a new event‐driven CBK data streaming network with Apache Kafka to enable researchers to do two tasks fast: (1) Create local codes for research needs and integrate them into UMLS standards codes; (2) Publish and subscribe to CBKs for real‐time clinical collaborations. We are looking for clinical researchers to address the urgent need of disseminating convid‐19 treatment knowledge from successful practices to clinics around the world, particularly in developing countries. Such CBK‐enabled semi‐automated clinical collaborations will save lives in fighting the current pandemic. We also plan to test CBK network to reduce health care disparities through facilitating rapid dissemination of rare disease CBK from advanced teaching hospitals to rural clinics.

## Employing hybrid reasoning to support clinical decision‐making

### Sabbir M. Rashid^1^, Morgan A. Foreman^2^, Daniel M. Gruen^1^, Jamie P. McCusker^1^, Oshani Seneviratne^1^, Amar K. Das^2^, Deborah L. McGuinness^1^

#### ^1^Rensselaer Polytechnic Institute, Troy, New York; ^2^IBM Research, Cambridge, Massachusetts

Hybrid reasoning, which involves the combination of multiple forms of reasoning in conjunction, is often used by physicians performing reasoning tasks involving clinical decision‐making. The Select and Test Model is an epistemological framework that represents how hybrid reasoning can be employed in a clinical setting. Based on this framework and by leveraging semantic technologies, we design and implement an AI system that can support healthcare providers with their reasoning tasks. We focus on reasoning tasks, such as differential diagnosis, treatment planning, and plan critiquing, considering strategies clinicians commonly use. By providing clinicians with an evidence‐based clinical decision‐support system, this work has the potential to improve patient care.

## How can we trust computable knowledge? Some UK perspectives

### Philip Scott^1^

#### ^1^University of Portsmouth, Hampshire, UK

Introduction: Several scandals involving poorly implemented algorithms have raised concerns about the safety and trustworthiness of computable knowledge, especially where there is some level of automation. The concept of open libraries of knowledge objects prompts new questions about clinician and public trust, content curation and regulation. We held a workshop in December 2019 to elicit clinician and patient views about assurance principles to build trust in next‐generation clinical decision support.

Methods: We recruited 14 participants, a mixture of clinicians and patients. The half‐day focus group opened with an introductory presentation explaining the concept of computable knowledge and highlighting some of the known risks. The discussion centred on questions of governance, priorities, concerns and evaluation.

Results: The discussion identified six key themes as crucial for trust: regulation, transparency, ease of use, confidence, evaluation and particular issues to resolve (multi‐morbidity and missing data). Regulatory concerns included “off‐label” algorithms, professional requirements in software engineering and safety‐critical design approaches.

Conclusions: There is clinical and public expectation of suitable controls and safety methods in the design, implementation and governance of computable biomedical knowledge.

## Identification and care coordination of child sex trafficking victims in the Emergency Department using BPM+ to translate knowledge into clinical decision support

### Katherine Ariano^1^, Ruben Medalla^1^, Alison Lieb^1^, Rachel Richesson^1^, Anna Orvola^2^, Lee Wise^3^, Ann Nguyen^4^

#### ^1^Duke University School of Nursing, Durham, North Carolina; ^2^Tufts University School of Medicine, Boston, Massachusetts; ^3^Clinch Valley Medical Center, Richlands, Virginia; ^4^University of Kentucky Healthcare, Lexington, UK

Numerous and constantly changing online information sources provide public health (PH) guidance about COVID‐19. Systematically inventorying online guidance is critical to understand what information is available, from where, and how it changes over time. We implemented an automated process to identify online COVID‐19 guidance resources (URLs). The structures of websites of state PH agencies for Florida (FL), Illinois (IL), Massachusetts (MA) and Utah (UT) were discovered using a commercial Web crawler. URLs identified via Web crawler were processed by scripts in Apple Automator to extract links to CDC resources from webpages and static documents. Inventoried URLs were screened for relevance. The automated process produced an inventory of guidance resources owned by a state PH authority; and an inventory of external guidance resources referenced by the state PH authority. Number of state‐maintained COVID‐19‐related URLs was 54 for FL; 216 for IL; 581 for MA; and 229 for UT. Of total URLs, guidance documents constituted 57% for FL; 51% for IL; 55% for MA; and 32% for UT. A random sample of guidance documents (n = 234) was drawn to support the development of a preliminary taxonomy of COVID‐19 PH guidance.

## Linking COVID‐19 public health guidance to MeSH terms

### Elisa Rocha^1^, Peter Taber^2^, Adria Lam^3^, Saifon Phengphoo^4^, Guilherme Del Fiol^5^, Catherine J. Staes^4^, Saverio M. Maviglia^6^, Roberto A. Rocha^6^

#### ^1^University of Massachusetts Medical School, Worcester, Massachusetts; ^2^ VA Salt Lake City Health Care System, Salt Lake City, Utah; ^3^Albany Medical College, Albany, New York; ^4^College of Nursing University of Utah, Salt Lake City, Utah; ^5^Department of Biomedical Informatics, University of Utah, Salt Lake City, Utah; ^6^Brigham and Women's Hospital, Harvard Medical School, Semedy, Inc., Boston, Massachusetts

Easy and consistent access to high‐quality information is critical nowadays considering the COVID‐19 pandemic. Stakeholders with diverse information needs use online sources to find guidance. In an effort to improve the “findability” of COVID‐19 information developed by public health agencies, we are creating a taxonomy to index and classify available guidance using an inductive methodology. Websites and documents authored by the CDC, the Utah Department of Health, and the Massachusetts Department of Public Health, are reviewed and coded. Identified concepts are defined and added to the taxonomy. Concepts are subsequently mapped to MeSH to corroborate their meaning and enable connections to other information sources. The ongoing effort to identify relevant MeSH terms has revealed gaps related to COVID‐19. Over 60% of the taxonomy concepts were successfully matched to current MeSH terms, approximately 17% had related terms but meaning nuances relevant to COVID‐19 were difficult to represent, and 2% of terms had no match. An example of a missing concept is “reopening procedure,” made important given the quarantine period. Promptly establishing comprehensive concept coverage is critical to timely and effective information retrieval during and after a pandemic.

## Provenance models can be extended to support computable clinical guidelines

### James P. McCusker ^1^, Henrique Santos^1^, Oshani Seneviratne^1^, Amar Das^2^, Deborah L. McGuinness ^1^

#### ^1^ IDEA, Rensselaer Polytechnic Institute, Troy, New York; ^2^ IBM, Cambridge, Massachusetts

Introduction: Provenance models can support representation of clinical guidelines by aligning them with variations on Deontic Logic's Traditional 3fold Classification.^1^ It is possible to designate subclasses of these groups as different kinds of activities, and then formally define the criteria for them using Web Ontology Language (OWL) property restrictions. Traditional permission models think in terms of Obligatory, Optional, and Impermissible activities, while medical guidelines are phrased as Recommended, Optional, and Discouraged. This was originally demonstrated in radio spectrum policy management and is currently being applied to the American Diabetes Association guidelines for Type II Diabetes management.

Methods: By providing property restrictions based on observations of current conditions, we can formulate assertions like “exercise is recommended when diabetic patients have blood pressure above this level” into ongoing attributes on people like current diagnosis and blood pressure, leading to recommended activities for that person.

Results: We present a representation for expressing computable guidelines using this approach with OWL.

Conclusion: Deontic and other modal logics can be represented and computed on within description logics implementations like OWL. This poster presentation is particularly relevant to people who are trying to determine how to guarantee a consistent interpretation of computable knowledge across platforms.

^1^ McNamara, Paul, “Deontic Logic”, The Stanford Encyclopedia of Philosophy (Summer 2019 Edition), Edward N. Zalta (ed.), https://plato.stanford.edu/archives/sum2019/entries/logic-deontic.

## Reducing HIT burden and improving outcomes

### Pawan Goyal^1^

#### ^1^ ACEP, Irving, Texas

Introduction: Emergency clinicians face increasing burdens: patients are new, unscheduled and high volume, the pattern of care is variable and almost unnavigable, data discovery is onerous, and hospital electronic medical record (EMR) design and usability does not do enough to facilitate efficient, high‐quality care. Rapid changes in recommended emergency clinician processes also require local EMR build teams to interpret and build updates to systems. Replicating this process across the hospitals creates redundant workflows.

The American College of Emergency Physicians (ACEP) aims to address clinician burden by fixing protocol design flaws. In this poster, ACEP demonstrates how it collaboratively converted a Clinical Policy and Best Practice into a digital protocol that is both machine and human readable.

Methods: The First Trimester Bleeding protocol was identified as ideal for protocol‐building as it is supported by an ACEP Clinical Policy American College of Obstetricians and Gynecologists (ACOG) guidance. Physicians directly involved in the writing of the corresponding policies, workflows and best practices provided subject matter expertise during the modeling processes. We used Business Process Management Plus (BPM+) to model the protocols as it has several ideal characteristics: human and machine readable, defined scope, market viability, scalability and cross‐specialty appeal.

Results: ACEP developed two branches of the protocol on based on patient inclusion criteria: Is the Patient Hemodynamically Stable or Unstable? The Stable Patient branch was fully developed with a differential diagnosis output requiring three key inputs: Pelvic Ultrasound (US) results, β‐HCG quantitative level & finding of Tissue at Cervical Os. Example calculation:

- Input: [Pelvic US reveals an IUP is viable] + [β‐HCG = 4000] + [Tissue at Cervical Os]
- Output: [Spontaneous Abortion] or [Heterotopic Pregnancy]

Conclusions: ACEP is continuing the development of two use cases: First Trimester Bleeding & Alternatives to Opioids in Low Back Pain. We are also targeting new use cases, especially those with cross‐specialty appeal and a focus on pandemic protocols (eg, COVID‐19 severity). Finally, ACEP is actively pursuing the implementation of developed use cases into EMR systems.

Acknowledgment: Steve Hasley, MD, OMG, Pittsburg, PA; Kenneth S. Rubin, University of Utah, Salt Lake City, Utah; Dhruv B. Sharma, MS, ACEP, Irving, TX; Susan B. Promes, MD, MBA, PennState Health; Sigrid A. Hahn, MD, MPH, Mount Sinai; Alexis LaPietra, DO, St. Joseph's Health; Reuben J. Strayer, MD, Maimonides Medical Center; Robert F. Lario, PhD, University of Utah.

## Supporting user‐centric explanation types for clinical reasoning

### Shruthi Chari^1^, Oshani Seneviratne^1^, Daniel M. Gruen^1^, Morgan A. Foreman^2^, Amar K. Das^2^, Deborah L. McGuinness ^1^

#### ^1^Rensselaer Polytechnic Institute, Troy, New York; ^2^ IBM Research, Cambridge, Massachusetts

Introduction: The proliferation of machine learning in critical applications like healthcare has renewed interest in explainable Artificial Intelligence (AI) models and systems. However, current support for explainability may not address specific end‐user needs that arise in actual use. To bridge this gap, we designed a semantic representation, informed by a literature review and user study, which system designers can leverage to connect explanation types to user needs.

Methods: We conducted a literature review in various domains to identify different explanation types and user goals they address (eg, for education, clarification, and exploration purposes). We then designed an ontology that includes system and user attributes and connects needs to the literature‐derived explanation types. We validated and refined this ontology through user studies involving a concrete example in guideline‐based healthcare.

Results: We identified semantic attributes needed for AI explainability design, including representing forms of knowledge and reasoning. User studies confirmed the need for multiple explanation types, including contrastive, counterfactual, case‐based, and contextual explanations in clinical reasoning, and informed our understanding of when each applies.

Conclusion: This poster is particularly relevant to system designers who may be able to leverage our ontology‐enabled infrastructure to build explainable AI systems in clinical decision support settings.

