# POSTER ABSTRACTS from Third Annual Public Meeting: Mobilizing Computable Biomedical Knowledge (MCBK 2020)

**DOI:** 10.1002/lrh2.10262

**Published:** 2021-02-28

In the abstract “Public health guidance for the COVID‐19 pandemic: A preliminary qualitative analysis,”^1^ the author order was incorrect. The correct author order follows:

Adria Lam, Peter Taber, Elisa Rocha, Saifon Phengphoo, Guilherme Del Fiol, Catherine J. Staes, Saverio M. Maviglia, Roberto A. Rocha.

We apologize for this error.

## References

[1] POSTER ABSTRACTS from third annual public meeting: Mobilizing computable biomedical knowledge (MCBK 2020) . Learn Health Sys, 2021;5:e10256. 10.1002/lrh2.10256 PMC8051353 33905465

